# Beat-to-beat cardiac repolarization lability increases during hypoxemia and arousals in obstructive sleep apnea patients

**DOI:** 10.1152/ajpheart.00760.2023

**Published:** 2024-03-01

**Authors:** Serajeddin Ebrahimian, Saara Sillanmäki, Salla Hietakoste, Antti Kulkas, Juha Töyräs, Raquel Bailón, David Hernando, Carolina Lombardi, Ludger Grote, Maria R. Bonsignore, Tarja Saaresranta, Jean-Louis Pépin, Timo Leppänen, Samu Kainulainen

**Affiliations:** ^1^Department of Technical Physics, University of Eastern Finland, Kuopio, Finland; ^2^Diagnostic Imaging Center, Kuopio University Hospital, Kuopio, Finland; ^3^Institute of Clinical Medicine, University of Eastern Finland, Kuopio, Finland; ^4^Department of Clinical Neurophysiology, Seinäjoki Central Hospital, Seinäjoki, Finland; ^5^School of Electrical Engineering and Computer Science, The University of Queensland, Brisbane, Australia; ^6^Science Service Center, Kuopio University Hospital, Kuopio, Finland; ^7^Biomedical Signal Interpretation and Computational Simulation (BSICoS) Group, Aragón Institute of Engineering Research (I3A), IIS Aragón, University of Zaragoza, Zaragoza, Spain; ^8^Centro de Investigación Biomédica en Red en Bioingeniería, Biomateriales y Nanomedicina (CIBER-BBN), Madrid, Spain; ^9^IRCCS Istituto Auxologico Italiano, Department of Cardiovascular, Neural and Metabolic Sciences, Milan, Italy; ^10^Department of Medicine and Surgery, University of Milano-Bicocca, Milan, Italy; ^11^Department of Sleep Medicine, Sahlgrenska University Hospital, Gothenburg, Sweden; ^12^Sleep and Vigilance Laboratory, Department of Internal Medicine, University of Gothenburg, Gothenburg, Sweden; ^13^PROMISE Department, University of Palermo and IRIB-CNR, Palermo, Italy; ^14^Division of Medicine, Department of Pulmonary Diseases, Turku University Hospital, University of Turku, Turku, Finland; ^15^Sleep Research Centre, Department of Pulmonary Diseases and Clinical Allergology, University of Turku, Turku, Finland; ^16^Inserm U1300, HP2 Laboratory, University of Grenoble Alpes, Grenoble, France

**Keywords:** beat-to-beat QT variability, nocturnal desaturation, obstructive sleep apnea, sleep arousal, ventricular repolarization

## Abstract

Obstructive sleep apnea (OSA) is associated with the progression of cardiovascular diseases, arrhythmias, and sudden cardiac death (SCD). However, the acute impacts of OSA and its consequences on heart function are not yet fully elucidated. We hypothesized that desaturation events acutely destabilize ventricular repolarization, and the presence of accompanying arousals magnifies this destabilization. Ventricular repolarization lability measures, comprising heart rate corrected QT (QTc), short-time-variability of QT (STVQT), and QT variability index (QTVI), were calculated before, during, and after 20,955 desaturations from lead II electrocardiography signals of 492 patients with suspected OSA (52% men). Variations in repolarization parameters were assessed during and after desaturations, both with and without accompanying arousals, and groupwise comparisons were performed based on desaturation duration and depth. Regression analyses were used to investigate the influence of confounding factors, comorbidities, and medications. The standard deviation (SD) of QT, mean QTc, SDQTc, and STVQT increased significantly (*P* < 0.01), whereas QTVI decreased (*P* < 0.01) during and after desaturations. The changes in SDQT, mean QTc, SDQTc, and QTVI were significantly amplified (*P* < 0.01) in the presence of accompanying arousals. Desaturation depth was an independent predictor of increased SDQTc (β = 0.405, *P* < 0.01), STVQT (β = 0.151, *P* < 0.01), and QTVI (β = 0.009, *P* < 0.01) during desaturation. Desaturations cause acute changes in ventricular repolarization, with deeper desaturations and accompanying arousals independently contributing to increased ventricular repolarization lability. This may partially explain the increased risk of arrhythmias and SCD in patients with OSA, especially when the OSA phenotype includes high hypoxic load and fragmented sleep.

**NEW & NOTEWORTHY** Nocturnal desaturations are associated with increased ventricular repolarization lability. Deeper desaturations with accompanying arousals increase the magnitude of alterations, independent of confounding factors, comorbidities, and medications. Changes associated with desaturations can partially explain the increased risk of arrhythmias and sudden cardiac death in patients with OSA, especially in patients with high hypoxic load and fragmented sleep. This highlights the importance of detailed electrocardiogram analytics for patients with OSA.

## INTRODUCTION

Obstructive sleep apnea (OSA) is a prevalent sleep disorder with a major global negative health impact, affecting an estimated population of 1 billion adults worldwide (1). OSA is estimated to affect 34% of men and 17% of women in the general population (2), and it is highly prevalent in patients with cardiovascular diseases (CVDs) (3). The current diagnosis and severity estimation of OSA are based on the average number of respiratory events per hour of sleep, i.e., the apnea-hypopnea index (AHI) (4). The AHI does not consider the impact of OSA on cardiovascular (CV) function, whereas evidence demonstrates a strong association between OSA and the progression of CVDs (5). Furthermore, OSA is associated with an increased risk of arrhythmia and sudden cardiac death (SCD) (6, 7). Studies have highlighted the potential of using the duration of apnea and hypopnea events and the severity of related desaturations as valuable markers for identifying patients with OSA with increased risk of cardiovascular morbidity and mortality (8–10). Despite an increasing body of evidence on the effects of OSA on cardiovascular function, the acute impacts of OSA and its consequences on heart function during repeated apneas and hypopneas are not fully explored. These acute effects might contribute to the association between OSA and adverse cardiac events including arrhythmia and SCD.

Impacts of OSA on the cardiovascular function are not fully elucidated, yet OSA has been associated with certain alterations in cardiovascular function. OSA can cause hypoxemia, hypercapnia, intrathoracic pressure swings, and arousals from sleep (3). On the other hand, hypoxemia and blood oxygen desaturations are associated with increased sympathetic discharges mediated by chemoreceptor reflexes (11). In addition, arousals may also exert transient surges in sympathetic activity (11). Alterations in autonomic function can lead to lability in the ventricular repolarization phase of the heart function (12) reflected in the T-wave of a surface electrocardiogram (ECG) signal. Moreover, hypoxia-induced chemoreceptor activation can promote hyperventilation, which can lead to abnormalities in ventricular repolarization (13). Markers of ventricular repolarization lability, including QT interval variability (with and without heart rate correction) and T-wave peak to T-wave end interval, are well established determinants for the risk of arrhythmias and SCD (14–16).

Sustained intermittent hypoxemia has been shown to destabilize ventricular repolarization, whereas the frequency of arousals does not seem to contribute to this destabilization (17). Conversely, another study (18) revealed that arousals have acute effects on ventricular repolarization lability. However, only one study has focused on the acute impact of nocturnal desaturations on ventricular repolarization (19), and a comprehensive understanding of the effects of desaturations on ventricular repolarization is lacking. Therefore, the current study aims to examine the acute impact of blood oxygen desaturations on beat-to-beat variability of ventricular repolarization parameters. We investigate how these parameters change during and after desaturations and whether they are affected by accompanying arousals, confounding factors, and common comorbidities of OSA. We hypothesize that desaturations increase ventricular repolarization parameter values, and the variations in these values are modulated by the severity of the desaturation events. Furthermore, the presence of accompanying arousals is further hypothesized to destabilize ventricular repolarization during desaturation.

## MATERIALS AND METHODS

### Data Set

In this retrospective study, the data set consisted of 917 consecutive type I polysomnography (PSG) recordings of patients with suspected OSA. The PSG recordings were conducted at the Princess Alexandra Hospital (Brisbane, Australia) between 2015 and 2017 with the Compumedics Grael acquisition system (Compumedics, Abbotsford, Australia). The PSGs were scored manually according to the American Academy of Sleep Medicine 2012 guidelines (4). All oxygen desaturation events with ≥3% blood oxygen saturation ($\text{S}{\text{p}}_{{\text{O}}_{2}}$) drop were scored from the onset of the event to the recovery of oxygenation. A detailed description of the scoring protocol is presented in our previous works (20, 21). Approval for the collection of retrospective data and its reuse was granted by The Metro South Human Research Ethics Committee, Brisbane, Australia (LNR/2019/QMS/54313). Due to the retrospective nature of the study, the need for informed consent was waived by the Metro South Human Research Ethics Committee.

Electrocardiogram (ECG) signals recorded during PSGs were manually reviewed, and subjects with insufficient ECG signal quality (*n* = 135) or apparent T-wave abnormalities (inverted T-wave, biphasic T-wave, and prominent U-wave, *n* = 121) were excluded from the study. Furthermore, we excluded patients having a pacemaker (*n* = 26), previous heart failure (*n* = 23) or respiratory failure (*n* = 25), atrial fibrillation/flutter (*n* = 41), and less than 4 h of objective sleep measured during PSG (*n* = 197). Given that some patients shared multiple exclusion criteria, the final number of excluded patients was 425 (Supplemental Fig. S1; all Supplemental material is available at https://doi.org/10.6084/m9.figshare.25288909). The final data set comprised 492 patients (Table 1). The characteristics of excluded patients and their comparison with included patients are presented in Supplemental Table S1.

**Table 1.** Characteristics of the study population

Clinical Characteristics	
Patients, *n*, (male%)	492 (52.0)
Age, yr	51.1 (40.5, 61.7)
BMI, kg/m^2^	33.7 (28.7, 39.4)
AHI, 1/h	14.3 (6.1, 29.3)
ArI, 1/h	24.6 (16.8, 37.4)
ODI, 1/h	12.3 (2.9, 30.1)
TST, min	334.5 (290.4, 373.9)
T90, min	1.9 (0.0, 21.6)
Comorbidities	
Arrhythmia history	19 (3.8)
COPD	32 (6.5)
Dys/hyperlipidemia	83 (16.8)
Hypertension	181 (36.8)
Stroke history	20 (4.1)
T2DM	81 (16.5)
Medications†	*n* = 277
Antipsychotics	15 (5.4)
β-blockers	45 (16.2)
Calcium channel blockers	42 (15.1)
Hypnotics	6 (2.2)
Other antiarrhythmics	0 (0)

Values are presented as number (%) or median (interquartile range) where appropriate. AHI, apnea-hypopnea index; ArI, arousal index; BMI, body mass index; COPD, chronic obstructive pulmonary disease; ODI, oxygen desaturation index; TST, total sleep time; T90, sleep time with oxygen saturation less than 90%; T2DM, diabetes mellitus type II. †Due to the lack of a complete list of medications for all patients, medication data are available only for a subpopulation.

### ECG Analysis

**Figure 1.**
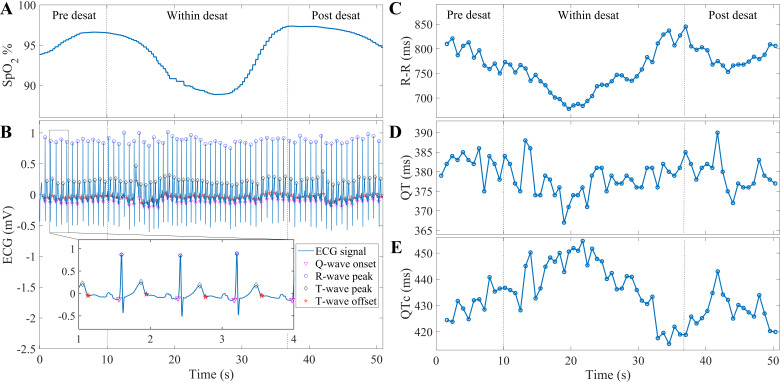
Demonstration of electrocardiogram (ECG) parameters for a desaturation event: detected desaturation event (*A*); delineated time-matched pre-, within-, and post-desaturation ECG samples (*B*); calculated RR, QT, and QTc interval time series (*C*–*E*, respectively). Desat, desaturation.

ECG signals were recorded with a modified lead II configuration with a sampling frequency of 256 Hz. ECG signals were filtered with a 4th-order Butterworth band-pass filter (0.05–40 Hz). For each desaturation event, we extracted three ECG samples based on the start and end times of desaturation events: a 10-s pre-desaturation sample prior to the onset of the desaturation, a sample during the entire desaturation, and a 15-s post-desaturation sample (Fig. 1). In cases where the post-desaturation and pre-desaturation samples of two consecutive desaturations overlapped, we excluded all samples from the latter desaturation from further analysis.

Each ECG sample was automatically delineated using a wavelet-based ECG delineator (22) and inspected for the presence of ectopic beats (23). ECG samples were upsampled to 1,000 Hz before delineation process. Segments containing an ectopic beat or having beat delineation rejection (beats could not be delineated due to the high noise) >20% were excluded to ensure accurate measurement of the parameters. Furthermore, we excluded the segments with mean heart rate (HR) <30 beats/min to exclude possible low-quality ECG samples. If one of the pre-, within-, or post-desaturation segments was omitted, the entire sequence was excluded. In total, 15,644 desaturations were excluded from the analysis.

After the exclusion of possible low-quality segments, 20,955 desaturations were considered in the analysis (Supplemental Fig. S1). The RR interval, the interval between T-wave peak and T-wave end (Tpe), the QT interval, and the HR-corrected QT interval (QTc) were calculated in a beat-to-beat manner for each ECG sample (parameters demonstrated in Fig. 1). The QTc was calculated based on the Bazett’s formula (24). Short-time variability of QT interval (STVQT) and QT interval variability index (QTVI) were calculated for each sample according to the following formula (16):

(*1*) $$\text{STVQT}=\overset{N-1}{\underset{n=1}{\sum }}\frac{\left|{\text{QT}}_{n+1}-{\text{QT}}_{n}\right|}{N\;\sqrt{2}}$$

(*2*) $$\text{QTVI}={\log\;}_{10}\frac{{\text{SDQT}}^{2}/{\text{QT}}_{\text{mean}}^{2}}{{\text{SDRR}}^{2}/{\text{RR}}_{\text{mean}}^{2}}\;$$

where *N* represents the number of beats in the segments and SD stands for standard deviation.

**Figure 2.**
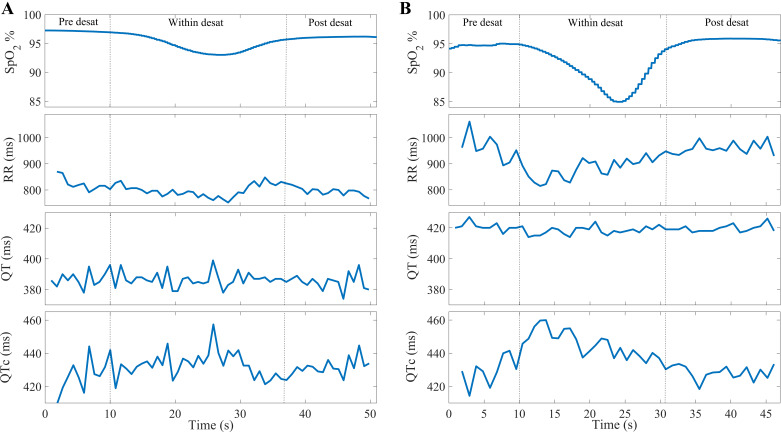
Calculated RR, QT, and QTc interval time series: a shallow desaturation without arousal (*A*) and a deep desaturation accompanied by an arousal (*B*). Deep desaturations accompanied by arousals lead to higher decrease in RR intervals and a notable increase in QTc values. Desat, desaturation; QTc, heart rate corrected QT interval.

Variations in the calculated parameters during the pre-desaturation sample were compared with that of the within-desaturation and post-desaturation samples to investigate the variations in ventricular repolarization during and after each desaturation in men and women. In addition, we studied whether the presence or absence of accompanying respiratory arousals (when there is overlap between occurrence of desaturation and arousal) affected the ventricular repolarization variations during and after desaturations. To assess the modulatory effects of desaturation severity, the data set was stratified into four groups based on both the duration (*T*_des_; 10 s ≤ *T*_des_ < 20 s, 20 s ≤ *T*_des_ < 30 s, 30 s ≤ *T*_des_ < 45 s, and *T*_des_ ≥ 45 s) and depth (3% ≤ Δ$\text{S}{\text{p}}_{{\text{O}}_{2}}$ < 4.5%, 4.5% ≤ Δ$\text{S}{\text{p}}_{{\text{O}}_{2}}$ < 6%, 6% ≤ Δ$\text{S}{\text{p}}_{{\text{O}}_{2}}$ < 7.5%, and Δ$\text{S}{\text{p}}_{{\text{O}}_{2}}$ ≥ 7.5) of desaturations (Table 2). Furthermore, we explored whether the occurrence of desaturation event in nonrapid eye movement (NREM) or rapid eye movement (REM) sleep is associated with changes in ventricular repolarization. Figure 2 demonstrates the calculated RR, QT, and QTc interval time series for a shallow desaturation (Fig. 2*A*) and a deep desaturation accompanied by an arousal (Fig. 2*B*).

**Table 2.** Number of analyzed desaturations in different subgroups based on duration and depth of desaturations

	Desaturation without an arousal (*n* = 10,622)	Desaturation with an arousal (*n* = 10,333)
Duration		
10 s ≤ *T*_des_ < 20 s	2,671	1,460
20 s ≤ *T*_des_ < 30 s	3,589	3,010
30 s ≤ *T*_des_ < 45 s	2,927	3,976
45 s ≤ *T*_des_	1,435	1,887
Depth		
3% ≤ Δ$\text{S}{\text{p}}_{{\text{O}}_{2}}$ < 4.5%	6,952	4,346
4.5% ≤ Δ$\text{S}{\text{p}}_{{\text{O}}_{2}}$ < 6%	1,200	1,129
6% ≤ Δ$\text{S}{\text{p}}_{{\text{O}}_{2}}$ < 7.5%	1,225	1,489
7.5% ≤ Δ$\text{S}{\text{p}}_{{\text{O}}_{2}}$	1,245	3,369

Statistical tests were performed using the chi-squared test. The difference between distributions of desaturations with and without accompanied arousals in both duration and depth groups is statistically significant. *T*_des_, desaturation duration; Δ$\text{S}{\text{p}}_{{\text{O}}_{2}}$, drop in the blood oxygen saturation.

Wilcoxon’s signed-rank test was used to analyze the statistical significance of the change from pre-desaturation to within- and post-desaturation values. For statistical significance between different severity groups, we assumed that the groups are not statistically independent as they could contain samples from the same patient. Therefore, we iteratively used Wilcoxon’s signed-rank test due to its pairwise comparison as described in our previous study (25). To identify the effects of potential confounding factors and comorbidities, we used a multiple regression model for analysis. In addition, due to the absence of a complete medication list for all patients in the studied population, a separate regression model was used to analyze the effects of possible confounding medications in a subpopulation of the data set. Statistical data analysis was conducted using MATLAB R2022b, with the limit for statistical significance set at *P* < 0.01.

## RESULTS

Oxygen desaturations caused significant (*P* < 0.01) changes in ventricular repolarization parameters during and after desaturations compared with the pre-desaturation values. The standard deviation of the QT time (SDQT), STVQT, and standard deviation of the Tpe (SDTpe) increased significantly (*P* < 0.01) during desaturations and remained at a higher level in post-desaturation compared with the pre-desaturation in men and women (Tables 3 and 4). However, mean QT and Tpe intervals did not change significantly during and after desaturations. Furthermore, desaturations caused a significant increase (*P* < 0.01) in both QTc and standard deviation of the QTc (SDQTc), meanwhile QTVI was observed to decrease (*P* < 0.01) during desaturations and increase during post-desaturations in men and women (Tables 3 and 4). In general, changes in ventricular repolarization parameters from baseline to within- and post-desaturations were greater when desaturations were accompanied by arousals. Ventricular repolarization parameter (mean QT, SDQT, mean QTc, SDQTc, STVQT, QTVI, and SDTpe) values were significantly higher in women compared with men (Tables 3 and 4). SDQT, SDQTc, STVQT, and SDTpe were significantly higher in NREM sleep compared with REM sleep in pre-, within-, and post-desaturation segments (Supplemental Table S2).

**Table 3.** Variations in ventricular repolarization parameters in pre-, within-, and post-desaturation segments in men

Parameter	Pre-desaturation	Within-desaturation	Post-desaturation
Desaturations without arousals (*n* = 6,716)			
Mean RR	888.6 (801.7, 1000.8)	**879.7 (780.7, 993.1)**	**876.4 (782.5, 989.0)**
SDRR	20.2 (12.4, 31.5)	**31.9 (20.0, 48.5)**	**24.8 (15.1, 38.3)**
Mean QT	398.5 (378.2, 425.2)	397.5 (377.7, 424.5)	398.2 (377.7, 424.7)
SDQT	4.22 (2.4, 9.7)	**6.1 (3.5, 15.9)**	**5.3 (3.0, 11.7)**
Mean QTc	423.4 (409.5, 438.8)	**426.6 (412.3, 441.7)**	**426.7 (412.2, 442.5)**
SDQTc	7.2 (4.4, 13.1)	**11.3 (6.9, 20.5)**	**9.3 (5.6, 17.1)**
STVQT	3.0 (1.7, 6.7)	**3.9 (2.2, 8.5)**	3.6 (2.0, 8.2)
QTVI	−1.4 (−2.8, 0.5)	−**1.6 (−3.1, 0.4)**	−1.4 (−2.9, 0.6)
Mean Tpe	82.7 (77.2, 92.9)	82.8 (77.4, 92.3)	82.8 (77.3, 92.8)
SDTpe	3.6 (2.1, 7.3)	**4.4(2.5, 14.0)**	**4.2(2.4, 9.4)**
Desaturations with arousals (*n* = 5,674)			
Mean RR	889.7 (810.6, 993.5)	871.2 (790.0, 966.6)*	**880.7 (795.7, 979.5)**
SDRR	22.5 (14.2, 35.6)*	50.3 (33.4, 72.3)*	31.1 (19.0, 49.6)*
Mean QT	402.6 (382.8, 426.7)*	402.6 (382.4, 424.7)*	402.4 (382.6, 426.1)*
SDQT	4.3 (2.5, 9.3)	7.1 (4.4, 18.0)*	**5.3 (3.1, 12.2)**
Mean QTc	426.3 (409.6, 445.0)*	430.7 (413.8, 451.8)*	428.7 (411.2, 450.8)*
SDQTc	7.6 (4.7, 13.6)	14.9 (10.0, 25.0)*	10.4 (6.2, 19.0)*
STVQT	3.0 (1.8, 6.6)	4.2 (2.3, 9.2)*	**3.5 (2.0, 8.0)**
QTVI	−1.7 (−3.0, 0.1)*	−2.3 (−3.4, −0.4)*	−1.9 (−3.2, 0.1)*
Mean Tpe	83.8 (77.2, 93.5)	83.5 (77.5, 93.5)	83.6 (77.3, 93.3)
SDTpe	3.9 (2.2, 8.3)	5.2 (2.9, 17.4)*	**4.3 (2.5, 10.5)**

Statistical tests were performed using the Wilcoxon signed-rank test. Values are presented as median (interquartile range). Bolded values indicate a statistically significant difference (*P* < 0.01) compared with the pre-desaturation value. Duration for all parameters except QTVI is presented in millisecond, QTVI is a dimensionless parameter. *Statistically significant difference compared with desaturation events without arousals. QTc, heart rate corrected QT interval; QTVI, QT variability index; SD, standard deviation; STVQT, short-time variability of QT; Tpe, interval between T-wave peak and T-wave end.

**Table 4.** Variations in ventricular repolarization parameters in pre-, within-, and post-desaturation segments in women

Parameter	Pre-desaturation	Within-desaturation	Post-desaturation
Desaturations without arousals (*n* = 3,617)			
Mean RR	857.4 (775.5, 971.8)†	**836.5 (762.3, 943.6)**†	**835.6 (759.7, 947.5)**†
SDRR	20.4 (12.7, 32.2)	**30.0 (19.4, 45.9)**	**24.2 (15.1, 38.6)**
Mean QT	410.3 (385.3, 435.3)†	409.6 (385.7, 434.6)†	410.1 (385.5, 434.7)†
SDQT	5.5 (3.1, 13.3)†	**7.9 (4.2, 22.8)**†	**6.8 (3.7, 22.7)**†
Mean QTc	436.8 (424.0, 454.9)†	**440.7 (428.2, 457.5)**†	**441.3 (428.4, 459.2)**†
SDQTc	8.6 (5.2, 17.6)†	**12.8 (7.8, 27.2)**†	**10.5 (6.3, 26.9)**†
STVQT	3.9 (2.3, 9.4)†	**5.3 (2.7, 12.1)**†	**4.8 (2.6, 12.5)**†
QTVI	**−**0.9 (**−**2.4, 1.0)†	**−1.0 (−2.5, 0.8)**†	**−**0.8 (**−**2.4, 1.0)†
Mean Tpe	83.2 (77.4, 94.4)	83.5 (77.5, 94.1)	83.7 (77.4, 94.8)
SDTpe	4.5 (2.7, 12.1)†	**5.7 (3.1, 22.3)**†	**5.3 (3.0, 22.0)**†
Desaturations with arousals (*n* = 4,948)			
Mean RR	872.9 (790.3, 974.8)*†	**847.6 (770.2, 936.8)**†	855.4 (775.1, 962.5)*†
SDRR	24.6 (15.7, 38.8)*†	50.0 (31.6, 74.5)*	32.6 (19.4, 52.6)*†
Mean QT	406.6 (392.0, 430.9)†	406.6 (391.6, 429.7)†	406.9 (391.1, 430.3)†
SDQT	5.5 (3.2, 12.3)†	9.5 (5.1, 24.8)*†	**6.9 (3.9, 20.2)**†
Mean QTc	434.4 (421.8, 450.6)*†	440.3 (429.1, 454.1)*†	437.9 (425.2, 453.6)*†
SDQTc	9.2 (5.6, 17.1)†	18.1 (10.4, 31.2)*†	**12.0 (7.2, 25.7)**†
STVQT	3.9 (2.2, 8.5)†	5.9 (2.9, 12.2)*†	**4.7 (2.6, 11.3)**†
QTVI	**−**1.3 (**−**2.7, 0.4)*†	−1.6 (−3.0, −0.1)*†	−1.4 (−2.8, 0.3)*†
Mean Tpe	83.5 (77.5, 92.6)	83.8 (78.1, 92.4)	83.7 (77.9, 93.0)
SDTpe	4.6 (2.8, 12.1)†	7.1 (3.6, 24.8)*†	**5.3 (3.1, 21.7)**†

Statistical tests were performed using the Wilcoxon signed-rank test. Values are presented as median (interquartile range). Bolded values indicate a statistically significant difference (*P* < 0.01) compared with the pre-desaturation value. Duration for all parameters except QTVI is presented in millisecond, QTVI is a dimensionless parameter. *Statistically significant difference compared with desaturation events without arousals. †Statistically significant difference compared with men (Table 3). QTc, heart rate corrected QT interval; QTVI, QT variability index; SD, standard deviation; STVQT, short-time variability of QT; Tpe, interval between T-wave peak and T-wave end.

**Figure 3.**
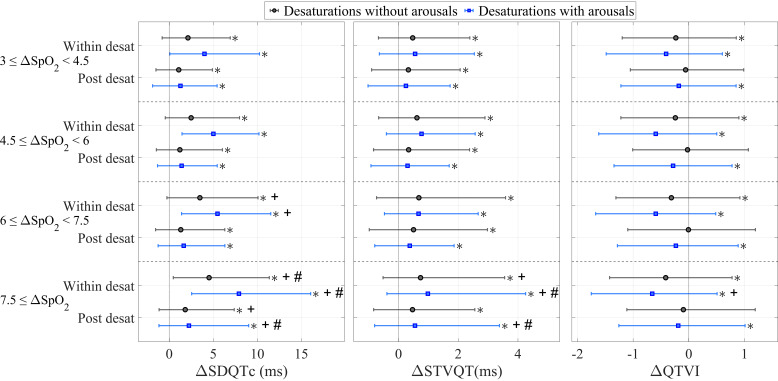
The changes in standard deviation of QTc (SDQTc), short-time variability of QT (STVQT), and QT variability index (QTVI) compared with the pre-desaturation measure between desaturations not associated with arousals and desaturations accompanied by arousals in different desaturation depth groups. Statistical tests were performed using Wilcoxon’s signed-rank test. Δ$\text{S}{\text{p}}_{{\text{O}}_{2}}$, blood oxygen desaturation in percentages; desat, desaturation. *Statistically significant change compared with the pre-desaturation values; +statistically significant change compared with the 3–4.5% group; #statistically significant change compared with all depth groups.

**Figure 4.**
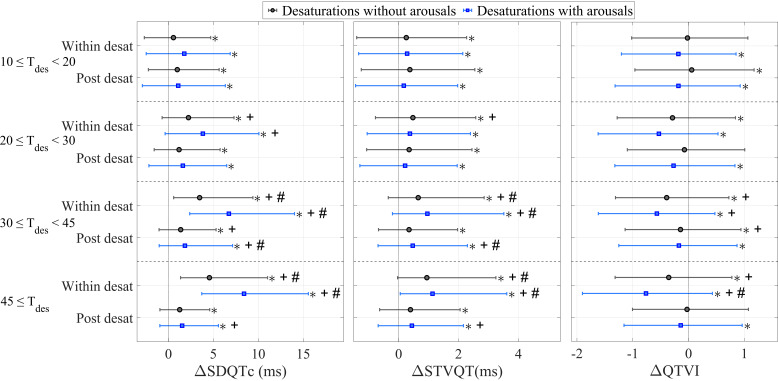
The changes in the standard deviation of QTc (SDQTc), short-time variability of QT (STVQT), and QT variability index (QTVI) compared with the pre-desaturation measure between desaturations not associated with arousals and desaturations accompanied by arousals in different desaturation duration groups. Statistical tests were performed using Wilcoxon’s signed-rank test. *T*_des_, desaturation duration in seconds; desat, desaturation. *Statistically significant change compared with the pre-desaturation values; +statistically significant change compared with the 10- to 20-s group; #statistically significant change compared with all duration groups.

The changes in SDQTc and STVQT in deep and long desaturations were significantly (*P* < 0.01) higher compared with less severe desaturations (Figs. 3 and 4). There were no significant differences in the changes in QTVI between different desaturation depth groups, whereas the changes were more notable between different desaturation duration groups (Figs. 3 and 4). Notably, accompanying arousals led to higher changes in SDQTc, STVQT, and QTVI during and after desaturations in all desaturation depth and duration groups (Figs. 3 and 4).

Regression analyses indicate that deepening of desaturations independently increases SDQTc, STVQT, and QTVI during desaturations after adjustment for pre-desaturation value, potential confounding factors, comorbidities, and medications (Table 5). The presence of accompanying arousal increased SDQTc, whereas decreased QTVI. In addition, occurrences of desaturations during NREM sleep independently increases SDQTc and STVQT. The type of the related respiratory events had not significant effect on parameters (Supplemental Table S3). However, related respiratory event duration and desaturation duration separately increased SDQTc and decreased QTVI, yet desaturation duration had higher impact on the parameter values (Table 5 and Supplemental Table S3). Both oxygen desaturation index (ODI) and sleep time with oxygen saturation less than 90% (T90) independently increased the values of all parameters (Supplemental Table S4). In contrast to our findings from comparing desaturation duration groups (Fig. 3), regression analysis indicated that the increase in desaturation duration was not linked with a significant change in STVQT.

**Table 5.** Generalized regression models for SDQTc, STVQT, and QTVI during desaturations

Model 1 (Confounding Factors and Comorbidities)						
Predictors	SDQTc		STVQT		QTVI	
	β-Coeff	*P* value	β-Coeff	*P* value	β-Coeff	*P* value
Age, yr	−0.008	0.259	**0.016**	**<0.01**	**0.007**	**<0.01**
BMI, kg/m^2^	−0.014	0.171	0.005	0.343	**0.004**	**<0.01**
Sex (male)	−**1.057**	**<0.01**	−**0.284**	**<0.01**	−**0.084**	**<0.01**
AHI, 1/h	0.005	0.139	−0.003	0.045	−**0.001**	**<0.01**
NREM sleep	**0.968**	**<0.01**	**0.356**	**<0.01**	0.060	0.028
Arousal	**1.741**	**<0.01**	−0.204	0.029	−**0.429**	**<0.01**
Pre-desaturation value, ms	**0.544**	**<0.01**	**0.595**	**<0.01**	**0.591**	**<0.01**
Desaturation duration, s	**0.077**	**<0.01**	0.006	0.034	−**0.008**	**<0.01**
Desaturation depth, %	**0.405**	**<0.01**	**0.151**	**<0.01**	**0.009**	**<0.01**
COPD	**1.034**	**<0.01**	0.250	0.117	**0.264**	**<0.01**
Hypothyroidism	**4.216**	**<0.01**	**2.046**	**<0.01**	**0.397**	**<0.01**
Hypertension	−**0.542**	**<0.01**	0.051	0.585	0.053	0.023
T2DM	−0.161	0.414	0.233	0.025	**0.120**	**<0.01**
Stroke history	**1.844**	**<0.01**	0.327	0.116	**0.157**	**<0.01**

Model 2 (Confounding Factors and Medications)						
Predictors	SDQTc		STVQT		QTVI	
	β-Coeff	*P* value	β-Coeff	*P* value	β-Coeff	*P* value
Age, yr	**0.063**	**<0.01**	**0.038**	**<0.01**	**0.001**	**<0.01**
BMI, kg/m^2^	**0.060**	**<0.01**	**0.028**	**<0.01**	−**0.004**	**<0.01**
Sex (male)	−**0.601**	**<0.01**	−**0.323**	**<0.01**	−**0.117**	**<0.01**
AHI, 1/h	0.005	0.108	−**0.008**	**<0.01**	−**0.001**	**<0.01**
NREM sleep	**1.135**	**<0.01**	**0.357**	**<0.01**	0.022	0.411
Arousal	**2.411**	**<0.01**	0.054	0.566	−**0.404**	**<0.01**
Pre-desaturation value, ms	**0.527**	**<0.01**	**0.590**	**<0.01**	**0.612**	**<0.01**
Desaturation duration, s	**0.075**	**<0.01**	0.004	0.144	−**0.012**	**<0.01**
Desaturation depth, %	**0.287**	**<0.01**	**0.101**	**<0.01**	**0.01**	**<0.01**
Antipsychotic	**3.024**	**<0.01**	**1.885**	**<0.01**	**0.250**	**<0.01**
β-blockers	−**1.797**	**<0.01**	−0.101	0.371	**0.281**	**<0.01**
Calcium blockers	**1.369**	**<0.01**	**0.691**	**<0.01**	**0.175**	**<0.01**

Parameters except desaturation duration, desaturation depth, and pre-desaturation values are treated patient wise; these parameters were computed for each desaturation separately. Boldface values indicate statistical significance (*P* < 0.01). AHI, apnea-hypopnea index; BMI, body mass index; β-Coeff, β-coefficient; COPD, chronic obstructive pulmonary disease; SDQTc, standard deviation of QTc, NREM, nonrapid eye movement; QTVI, QT variability index; STVQT, short-time variability of QT; T2DM, diabetes mellitus type II.

Among possible comorbidities, hypothyroidism was significantly associated with increase in SDQTc, STVQT, and QTVI, whereas chronic obstructive pulmonary disease (COPD) and a stroke history only affected SDQTc and QTVI (Table 5). The regression model for considering the effects of medication revealed that a history of using antipsychotic and calcium channel blockers increased the values of all parameters. On the other hand, a history of using β-blockers decreased SDQTc, whereas increased QTVI, with no significant effect on STVQT.

## DISCUSSION

This study examined the variations in the ventricular repolarization phase of cardiac cycle during and after sleep apnea-related desaturations. The present results indicate, as we hypothesized, that desaturations impose acute changes to beat-to-beat variation of ventricular repolarization during and after desaturation compared with the baseline reflected by an increase in mean QTc, SDQTc, and STVQT and yet a paradoxical decrease in QTVI. The changes resulting from desaturations were magnified by the presence of accompanying arousal according to our hypothesis. Further analysis revealed that desaturation depth was associated with an increased beat-to-beat ventricular repolarization independent of age, sex, body mass index (BMI), AHI, comorbidities, and medications. Our results provide evidence of the detrimental effects of nocturnal desaturations on ventricular repolarization and highlight the significance of utilization of detailed ECG waveform analytics in OSA diagnostics and risk stratification.

Hypoxia influences the heart function, particularly in the context of OSA. OSA-related hypoxemia has been shown to be associated with an increased risk of cardiovascular mortality (9). Elevated pulmonary artery pressure and chemoreceptor activation triggered by OSA induced acidosis, hypoxia, and hypercapnia, leading to both chronic and acute activation of the sympathetic autonomic nervous system (26). Previous research by Tavares et al. (26) demonstrated a progressive increase in sympathetic activation due to the decrease in blood oxygen saturation levels. Furthermore, acute hypoxia in animal models has been reported to alter cardiac ion channel functions and regulates the L-type Ca^2+^ channel by increasing their sensitivity to β-adrenergic stimulation, which in turn could lead to Ca^2+^ influx and prolongation of the action potentials (27). With elevated sympathetic activity and related increase in catecholamines caused by OSA, hypoxia could lead to cardiac instability. Solhjo et al. (17) reported that greater QTVI is independently associated with higher overall hypoxic load (T90) values and QTVI increases after sustained intermittent hypoxemia. Similar to the previous findings, our study also indicates that parameters related to overall hypoxic load (ODI and T90) are independent predictors of increased ventricular repolarization instability (Supplemental Table S4). In addition to that, our study provides more insights and novel information into the transient impacts of acute hypoxia on ventricular repolarization. A previous study indicated that the severity of desaturation events are associated with increased QTc, and the increase is even higher when the sympathetic activity is more pronounced due to previous stroke (19). Prolonged QTc interval and increased QTVI values have been linked to higher risks of ventricular arrhythmias, sudden cardiac death, and cardiovascular mortality (28, 29), and increased STVQT has been associated with an increased risk of arrhythmia (30). In the present study, we observed decreased QTVI during desaturation, and as hypothesized, increased mean QTc, SDQTc, and STVQT. The paradoxical transient decrease in QTVI could be due to difference in size of pre-desaturation and within-desaturation segment; calculation of QTVI values over longer segments with more samples are more stabilized hence might lead to systematically lower QTVI values for within-desaturation segments. Moreover, our analysis revealed that deeper desaturations independently increase the values of parameters reflecting beat-to-beat ventricular repolarization, including QTVI (Table 5). It is noteworthy that the impact of desaturation depth on ventricular repolarization was stronger than that of AHI, ODI, and T90, rendering it a more significant predictor of altered ventricular repolarization. Our results suggest that the risk of arrhythmias and SCD may be increased during severe desaturations in patients with OSA.

Nocturnal respiratory event cascades can involve both desaturations and arousals. Arousals are known to be accompanied by excessive sympathetic outflow (31), which in turn can increase QT variability and arrhythmogenicity. Moreover, the accumulated chemical drive caused by airway obstruction can lead to hyperventilation after arousal (32), which can further induce ventricular repolarization abnormalities (13). A previous study demonstrated that arousals have acute effects on ventricular repolarization and are accompanied by decreased QTVI (18). We explored whether arousals impose further changes in ventricular repolarization when they coincide with desaturations. Our results revealed that accompanying arousal magnifies the changes in ventricular repolarization in men and women (Tables 3 and 4), which can further increase the risk of arrhythmia and SCD. Moreover, the presence of arousal independently increased SDQTc and yet further decreased QTVI (Table 5). This further decrease in QTVI could be due to the transient increase in RR intervals before and after the onset of arousals (33). Our observations of the changes in QTVI are in line with the previous study (18). Meanwhile, desaturation depth increased QTVI independent of the presence of accompanying arousals (Table 5). Interestingly, excessive QTVI during arousals predicted all-cause and cardiovascular mortality (18). Therefore, arousals accompanied by deep desaturations can lead to an increased risk of malignant cardiac events. However, it is important to note that our analysis solely focused on respiratory arousals and their co-occurrence with desaturations. We explored potential differences in ventricular repolarization changes when arousal occurred in relation to a desaturation event. Further investigations are warranted to examine the beat-to-beat dynamics of ventricular repolarization during the typical sleep apneic cascades comprising the breathing cessation, increased respiratory effort, hypoxemic period, arousal, and the hyperventilation period. This could provide more comprehensive understanding of these complex interactions.

Several confounding factors are independently associated with increased ventricular repolarization instability. Our findings revealed that desaturations during REM or NREM sleep, as well as the sex of the individual, have a significant impact on the ventricular repolarization variations. Interestingly, the AHI was either insignificant or inversely related to increased ventricular repolarization instability. Based on previous evidence, women exhibit longer QTc intervals and higher QTVI values (34, 35), and in line with these findings, results demonstrated that ventricular repolarization values are significantly higher in women compared with men in pre-, within-, and post-desaturation segments regardless of presence of arousals (Table 4). Moreover, our regression analysis revealed that the sex-specific differences in ventricular repolarization are independent of confounding factors, comorbidities, and medication (Table 5). These sex-specific differences may be attributed, among other factors, to sex hormones (34). In addition, we observed that desaturations in NREM sleep were accompanied by significant increases in ventricular repolarization lability in men and women, but the absolute differences were small. Moreover, further analysis revealed that occurrence of desaturations in NREM sleep were independently associated with increased SDQTc and STVQT with insignificant effects on QTVI (Table 5). It has been reported that QTc intervals progressively increased toward deeper sleep stages in patients with OSA (36), and mean QTc intervals have been shown to be longer in NREM sleep for men and women (35). Moreover, differences in QTVI have been reported to be insignificant between NREM and REM sleep (35). The observed changes in ventricular repolarization during NREM sleep may be attributed to the elevated sympathetic modulation in patients with OSA in NREM sleep (37). However, further investigations are required to explore the specific variations of ventricular repolarization across different sleep stages and sleep cycles.

Certain drugs and comorbidities can affect ventricular repolarization. We found that hypothyroidism independently increased all measured parameters, whereas a history of stroke and COPD independently increased SDQTc and QTVI (Table 5). This is in line with previous studies showing that patients with hypothyroidism have elevated QTVI values (38) and individuals with COPD and stroke histories exhibited increased QTc intervals (19, 39). Furthermore, our analysis revealed significant confounding effects of antipsychotic and calcium channel blocker medications: the patients on these medications had increased ventricular repolarization lability during desaturations (Table 5). A history of using β-blockers had mixed effects on the parameters yet it indicates increased QTVI during desaturations.

Our study is not without limitations. We acknowledge the potential impacts of artifacts and noise on ECG delineations and parameter calculations. Due to the fully automated beat delineation process, occasional incorrect beat delineations may occur in the analyzed segments as a result of these artifacts. However, we took measures to monitor signal quality, identify T-wave abnormalities, and exclude segments with noisy beats to minimize the likelihood of including such artifacts in the analysis. Moreover, in our analysis, QTVI measures were calculated over shorter segments compared with recommended durations. However, calculation of QTVI over short segments and predictive value of short-time QTVI values for risk stratification have already been established (40, 41), and longer segments are not fully representative of the studied phenomena and interactions. Thus, we speculate that in the context of OSA and rapid dynamics of changes it causes, calculation of QTVI over short segments could provide insights regarding the effects of desaturation and arousals on ventricular repolarization. Yet we speculate the calculation of QTVI over segments with different sizes between pre-desaturation and within- or post-desaturation have led to paradoxical transient decrease in QTVI. The QTVI over longer segment and more data samples is more stabilized, leading to lower values compared with shorter segments. Furthermore, due to the retrospective nature of our study, some information regarding the studied population was missing. Complete medication lists were missing in some patients; however, we addressed the potential effects of confounding medications in a subpopulation with complete medical information to enhance our understanding. Nevertheless, the data set lacks information on electrolyte levels like serum potassium among patients. In addition, we conducted analyses considering comorbidity and medication data as dichotomous variables. We acknowledge that comorbidities such as hypertension vary in severity, and different medications can have a dose-dependent impact on cardiac electrophysiology. However, we believe that these considerations do not jeopardize our results. Furthermore, our data set lacks a clinical end point and due to methodological differences between studies, our results cannot directly provide information regarding the probability of CV incidents. However, previous studies have consistently connected increased QT variability measures to increased cardiovascular mortality and SCD (15, 28), and the predictive value of increased short-time STVQT and QTVI for incidences of major adverse cardiovascular events has been demonstrated in a large pool of subjects with no CVD (41). However, QT variability during desaturations and its direct associations with the risk of arrhythmia and SCD warrant further studies.

### Conclusions

In conclusion, nocturnal desaturation events in patients with OSA have acute effects on ventricular repolarization phase of the cardiac cycle, and the accompanying arousals magnify these effects. Moreover, deeper desaturations were independently associated with increased ventricular repolarization lability. These findings further raise the question of whether the frequent occurrence of profound desaturation events accompanied by arousals could serve as a risk marker for cardiovascular events is patients with OSA. In such cases, this could aid in identifying patients who would benefit from more comprehensive cardiac evaluation.

## DATA AVAILABILITY

Data cannot be shared publicly because of potentially identifying or sensitive patient information. These ethical restrictions are imposed by The Metro South Human Research Ethics Committee, Brisbane, Australia. Data are available from The Metro South Human Research Ethics Committee, Brisbane, Australia (contact via MSHEthics@health.qld.gov.au) for researchers who meet the criteria for access to confidential data. Researchers can contact the MSHEC and the project steering committee will review the requests.

## SUPPLEMENTAL DATA

10.6084/m9.figshare.25288909 Supplemental Fig. S1 and Supplemental Tables S1–S4: https://doi.org/10.6084/m9.figshare.25288909.

## GRANTS

This work was supported by the European Union’s Horizon 2020 Research and Innovation programme (965417 to T.L.); NordForsk (90458 to T.L. and J.T.) via Business Finland (5133/31/2018 to T.L. and J.T.); Research Committee of the Kuopio University Hospital Catchment Area for the State Research Funding (projects 5041790 to S.H., 5041794 to T.L., 5041798 to S.S., 5041804 to S.K., 5041808 to S.E.); Seinäjoki Central Hospital (7746 to A.K.); the Competitive State Research Financing of Expert Responsibility Area of Tampere University Hospital (VTR7319 to A.K., VTR7312 A.K., VTR7330 A.K., and EVO2089 to A.K.); Päivikki & Sakari Sohlberg Foundation (to S.H.); The Research Foundation of the Pulmonary Diseases (to S.E.); Finnish Cultural Foundation - Central fund (to S.K.); Tampere Tuberculosis Foundation to (S.K.); and Alfred Kordelin foundation (to S.H.). This work was partly supported by CIBER in Bioengineering (to R.B.). This work was also partly supported by CIBER in Bioengineering, Biomaterials & Nanomedicne (CIBERBBN) through Instituto de Salud Carlos III and FEDER (Spain), projects PID2021-126734OB-C21 funded by MICINN and FEDER to R.B., and Gobierno de Aragon (Reference Group BSICoS T39-23 R) cofunded by FEDER 2014–2020 “Building Europe from Aragon” (to R.B.). J.-L.P is supported by the French National Research Agency in the framework of the “Investissements d’avenir” program (ANR-15-IDEX-02) and the “e-health and integrated care and trajectories medicine and MIAI artificial intelligence” chairs of excellence from the Grenoble Alpes University Foundation. This work was partially supported by MIAI @ Grenoble Alpes, (ANR-19-P3IA-0003 to J.-L.P).

## DISCLOSURES

No conflicts of interest, financial or otherwise, are declared by the authors.

## AUTHOR CONTRIBUTIONS

S.E., S.S., and S.K. conceived and designed research; S.E. and S.K. analyzed data; S.E., S.S., S.H., A.K., J.T., R.B., D.H., C.L., L.G., M.R.B., T.S., J.-L.P., T.L., and S.K. interpreted results of experiments; S.E. prepared figures; S.E. drafted manuscript; S.S., S.H., A.K., J.T., R.B., D.H., C.L., L.G., M.R.B., T.S., J.-L.P., T.L., and S.K. edited and revised manuscript; S.S., S.H., A.K., J.T., R.B., D.H., C.L., L.G., M.R.B., T.S., J.-L.P., T.L., and S.K. approved final version of manuscript.
